# Laparoscopic Ureteroureterostomy vs. Common Sheath Ureteral Reimplantation in Children With Duplex Kidney Anomalies

**DOI:** 10.3389/fped.2021.637544

**Published:** 2021-02-18

**Authors:** Tim Gerwinn, Ralph Gnannt, Daniel M. Weber, Rita Gobet, Luca Mazzone

**Affiliations:** ^1^Division of Pediatric Urology, University Children's Hospital Zurich, Zurich, Switzerland; ^2^Children's Research Center, University Children's Hospital Zurich, Zurich, Switzerland; ^3^Department of Diagnostic Imaging, University Children's Hospital Zurich, Zurich, Switzerland

**Keywords:** ureteral reimplantation, ureteroureterostomy, laparoscopy, duplex kidney, pediatric

## Abstract

**Purpose:** Laparoscopic ureteroureterostomy (LUU) has been proposed as an alternative to common sheath ureteral reimplantation (CSUR) in children with symptomatic duplex kidneys. However, data is limited for LUU in the pediatric population. The aim of this study was to analyze our experience with LUU and to compare the results with those after CSUR to assess whether a less invasive surgical approach could be a valid alternative.

**Patients and methods:** The data of all children with duplex kidneys who underwent either LUU or CSUR at our center from 2006 to 2018 were reviewed retrospectively. After parental counseling, the option of LUU was provided as an alternative to CSUR for unilateral procedures and in the absence of vesicoureteral reflux to the receiving ureter. Baseline characteristics, indication for surgery, hospitalization and operative times, and intraoperative, post-operative, and late complications were analyzed. Preoperative and 1-year post-operative sonographies were reviewed by a pediatric radiologist. Increasing renal pelvic diameter (Δ >5 mm) was regarded as a sign of ureteral obstruction.

**Results:** Forty children were included in this study, with 16 children receiving LUU and 24 children receiving CSUR. The children had a mean age of 2.7 years (7 months−9.8 years) and were followed up in our outpatient clinic for an average of 3.9 years (3 months−10.6 years) after surgery. The median hospital stay was 2 days shorter after LUU. Initially, a considerably longer time was needed for LUU, but after more experience was gained, similar operative times were observed for both procedures. Complications were encountered in both groups. After LUU, two patients developed anastomotic leakage: one was managed conservatively, and one required temporary nephrostomy. In the CSUR group, one patient developed vesicoureteral obstruction during follow-up and required reoperation with LUU. The occurrence of post-operative urinary tract infections was similar in both groups. No complications related to the ureteral stump after LUU arose.

**Conclusion:** LUU is a safe and efficacious treatment option for children with duplex kidney anomalies and can be used as an alternative to CSUR. All children receiving LUU showed a non-obstructive, patent anastomosis and no signs for stenotic compromise of the receiving ureter.

## Introduction

Duplex kidney is the most common renal abnormality in children (1–3). Although its prevalence is high (up to 1% of the population) (4–6), medical problems requiring treatment are rarely encountered (7). However, associated pathologies require attention. Ectopic insertion of the upper pole ureter, ureteroceles, and vesicoureteral reflux (VUR) may cause renal damage due to obstruction and/or urinary tract infection (UTI) (8). Furthermore, incontinence in ectopic ureters and obstruction of the bladder neck by large ureteroceles can occur.

The aims of the surgical treatment are preservation of renal function by relieving obstruction and preventing UTIs or attainment of urinary continence in ectopic inserting ureters. Surgical strategies encompass procedures for acute decompression (nephrostomy, cutaneous ureterostomy, and transurethral ureterocele incision) and delayed reconstruction procedures (ureteroureterostomy or common sheath ureteral reimplantation). Lastly, in cases with non-functioning moieties, heminephrectomy may be considered (9, 10).

Although ureteroureterostomy, whether open or laparoscopic, has been described as a valid alternative to reimplantation, it is still not used routinely in many centers, and provided literature is limited by small case numbers (11–16). At our center, laparoscopic ureteroureterostomy (LUU) was introduced in 2006 after experience had been gained in other laparoscopic techniques in infants.

The aim of this study was to analyze our experience with LUU as a treatment option for children with duplex kidney anomalies and to compare it with the most widely used surgical treatment, the common sheath ureteral reimplantation (CSUR). Focus was put on efficacy, post-operative outcome, and surgical learning curve.

## Materials and Methods

For this retrospective study, we included all patients with duplex kidneys who underwent LUU at our center between 2006 and 2018. Patients with duplex kidneys undergoing CSUR during the same period were used as controls. CSUR represents the established and most performed procedure for patients with symptomatic duplex kidneys. The use of CSUR as control group is not based on surgical technicalities but, rather, on clinical application and practice, as both LUU and CSUR fix the same problem. During the pre-operative outpatient visit, LUU was presented to the child's parents as an alternative to CSUR for unilateral procedures and in the absence of VUR to the receiving ureter. After providing detailed information on both operative approaches, we left the decision on the choice of method to the parents. To avoid selection bias, all patients with CSUR were included despite the resulting unbalanced group size. Surgery-related codes and a full-text search in our electronic data management system were used to identify patients. Relevant data were collected from the patients' files. This included age at time of surgery, sex, indication for surgery, laterality of surgical site in case of LUU ipsilateral or translateral anastomosis, intraoperative and post-operative complications, time of hospitalization, and operative time. Pre-operative and 1-year post-operative sonographies with measurement of the renal pelvic diameter were retrospectively reassessed and re-evaluated by a specialist pediatric radiologist (RG). A post-operative increasing renal pelvic diameter (Δ >5 mm) was regarded as a sign of ureteral obstruction. The study was approved by the Ethical Committee of the Canton of Zurich (2019-00305).

### Operation Techniques

LUU was performed with a 5-mm camera port and two 3-mm working ports. Before laparoscopy began, a ureteral stent was inserted cystoscopically into the receiving ureter. The end-to-side anastomosis was performed as described by González et al. (11) at the level of the ureteral crossing of the iliac vessels. Resection of the ureteral stump was carried out as low as possible. Care was taken not to compromise the vascular supply of the receiving ureter. The anastomosis was performed with a running 5–0 monofilament absorbable suture. Before completion of the anastomosis, the proximal end of the ureteral stent was slightly retracted and then pushed over the anastomosis into the donor ureter and corresponding moiety. All patients received perioperative intravenous antibiotic prophylaxis with cefuroxime for 48 h and oral antibiotic prophylaxis with trimethoprim/sulfamethoxazole until cystoscopic ureteral stent removal 6–8 weeks post-operatively. Standardized sonographic follow-up occurred post-operatively at 3, 12, and 24 months and every 4 years thereafter.

CSUR was performed as described by Cohen or by Politano-Leadbetter. Ureteral stents were left in place in 16 patients but omitted in eight patients in whom a distinct urine jet was observed intraoperatively, and therefore vesicoureteral obstruction due to swelling was unlikely. All patients received perioperative intravenous antibiotic prophylaxis with cefuroxime for 48 h and oral antibiotic prophylaxis with trimethoprim/sulfamethoxazole for 3 months. Standardized sonographic follow-up occurred post-operatively at 3, 12, and 24 months and every 4 years thereafter.

No routine post-operative voiding cystourethrograms (VCUGs) were planned. At our center, VCUGs are only scheduled after recurrent UTIs, not for single events.

## Results

**Figure 1:**
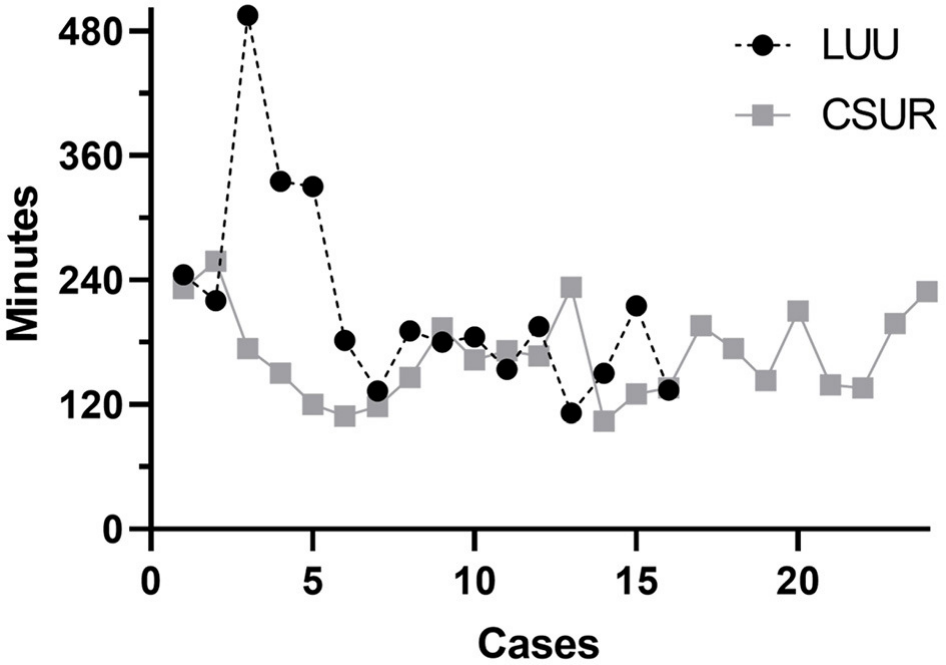
The operative times for both procedures gradually converge. Cases 3 and 4 in the laparoscopic ureteroureterostomy group were salvage procedures after failed common sheath ureteral reimplantations with anastomosis to the contralateral ureter and a bilateral procedure, respectively. Operative time was therefore significantly longer than in standard cases.

Sixteen patients had a LUU (2 male, 14 female), and the mean age at surgery was 2.2 years (7 months−9.8 years). The mean post-operative follow-up was 3.8 years (3 months−9.3 years). One patient was lost to follow-up (relocation to another country). Hospitalization time was 2–26 days, with a mean of 6.8 days and a median of 5 days. The operative time including cystoscopy was 112–495 min, with a mean of 216 min and a median of 188 min (Figure 1). Indications for LUU were ectopic ureter insertion in 10 patients (62.5%), causing obstruction in eight and incontinence in two patients. Four patients presented with recurrent febrile UTI due to VUR. Three patients had primary VUR to the lower moiety (18.8%), and one patient developed iatrogenic VUR after ureterocele incision (6.2%). In two patients (12.5%), LUUs were performed as salvage operation after failed CSURs due to post-operative vesicoureteral obstruction. Nine were left-sided procedures, six were right-sided, and one was bilateral. Anastomosis was performed 15 times to the ipsilateral ureter and once to the contralateral ureter (Table 1). There were no intraoperative complications. No patient required conversion to open surgery. Two patients (12.5%) suffered anastomotic leaks: one of these patients required a percutaneous nephrostomy, and the other was treated with bowel rest and intravenous antibiotics for paralytic ileus, urinoma, and UTI. Four (25%) post-operative febrile UTIs occurred during follow-up; all were single events, so we omitted invasive diagnostics by using VCUGs. The 1-year post-operative ultrasound was available in all but two patients. It showed a stable (Δ <5 mm) or decreasing renal pelvic diameter, thus showing no signs of post-operative obstruction. In two patients, only 3-month and 2-year post-operative ultrasounds were available. Those showed no signs of obstruction.

**Table 1:** Patient characteristics and summary of results [operative time: the statistical outliers causing the difference between mean and median in the laparoscopic ureteroureterostomy (LUU) group were one case of bilateral anastomosis (operative time, 335 min) and one case of contralateral LUU (operative time, 495 min); hospitalization: the statistical outliers causing the difference between mean and median in the LUU group were two cases with anastomotic leak (hospitalization time, 13 and 26 days)].

**Parameters**	**Laparoscopic ureteroureterostomy (*n* = 16)**	**Common sheath ureteral reimplantation (*n* = 24)**
**Demographics**		
Sex (male; female)	2; 14	4; 20
Mean age (range)	2.2 years (7 months−9.8 years)	3.1 years (10 months−9.3 years)
Mean follow-up (range)	3.8 years (3 months−9.3 years)	4.4 years (11 months−10.6 years)
**Indication**		
Febrile urinary tract infection (UTI) and vesicoureteral reflux (VUR)		
Primary VUR	3 (18.8%)	14 (58.3%)
Secondary VUR	1 (6.2%)	7 (29.2%)
Ectopic insertion		
Obstruction	8 (50%)	3 (12.5%)
Incontinence	2 (12.5%)	-
Salvage procedure	2 (12.5%)	-
**Side of procedure**		
Right	6	7
Left	9	5
Bilateral	1	12
**Anastomosis**		
Ipsilateral	15	
Contralateral	1	
**Operative time (min)**		
Range	112–495	104–258
Mean	216	169
Median	188	167
**Hospitalization (day)**		
Range	2–26	3–10
Mean	6.8	6.6
Median	5	7
**Complications**		
Intraoperative	-	-
Postoperative	2 (12.5%) anastomotic leaks with UTI	-
Follow-up	4 (25%) UTI	5 (20.8%) UTI, 1 (4%) obstruction

Twenty-four patients received a CSUR (4 male, 20 female), and the mean age at surgery was 3.1 years (10 months−9.3 years). The mean post-operative follow-up was 4.4 years (11 months−10.6 years). One patient was lost to follow-up (relocation to another country). The hospitalization time was 3–10 days, with a mean of 6.6 days and a median of 7 days. The operative time was 104–258 min, with a mean of 169 min and a median of 167 min (Figure 1). Indications for CSUR were recurrent febrile UTI in 14 patients (58.3%) with primary VUR to the lower moiety (one combined lower/upper moiety), iatrogenic VUR after ureterocele incision in seven patients (29.2%), and ureteral obstruction (two ureteroceles and one vesicoureteral obstruction) in three patients (12.5%). CSUR was performed in 12 cases unilaterally (seven right, five left) and in 12 cases bilaterally (Table 1). There were no intraoperative complications. Five patients (20.8%) suffered febrile UTI during follow-up; all were single events, so we omitted invasive diagnostics with VCUGs. One patient (4%) showed increasing renal pelvic diameter and required reoperation with LUU due to post-operative vesicoureteral obstruction. Apart from that, the 1-year post-operative renal pelvic diameter was stable (Δ <5 mm) or decreased in all patients; there were no further signs for post-operative vesicoureteral obstruction.

Due to poor image quality, post-operative renal diameter assessment was not possible in two patients with LUUs and four with CSURs.

## Discussion

Our data demonstrate that LUU is a safe and efficacious treatment option for selected patients with duplex kidney anomalies and thus can be used as a minimally invasive alternative to CSUR. All patients receiving LUUs showed a non-obstructive, patent anastomosis and, most importantly, no signs of stenotic compromise of the receiving ureter. Furthermore, none of the patients suffered complications related to the ureteral stump. However, complications after ureteroureterostomy such as anastomotic leaks, recurring febrile UTIs, ureteral strictures, worsening of hydronephrosis, reoperation on the distal ureter stump due to infection, and new-onset reflux have been described (17, 18). Lashley et al. (19) reported on 100 open ureteroureterostomies in children and described failure because of obstruction in three cases (3%), reflux in two (2%), and a non-draining ureteral stump in one (1%). Lee et al. (20) identified infections in the remnant ureteral stump requiring re-operation in 12% in their series of 74 adult patients. Michaud et al. (18) reviewed the available literature regarding complications in LUU and robot-assisted LUU in pediatric patients. A total of 51 cases had an overall complication rate of 7.8%, all with febrile UTIs and one reoperation for ureteral stent exchange. There was no reported case of post-operative obstruction, ureteral stricture, new-onset reflux, or ureteral stump excision (18). It is quite plausible that ureteral stump complications are less frequent when ureteroureterostomy is performed laparoscopically because the ureteral stump is likely to be left shorter when dissected laparoscopically.

Our LUU cohort included two patients (12.5%) with anastomotic leakage and consecutively prolonged hospitalization. Both patients presented with febrile UTI and paralytic ileus. Both patients were treated with antibiotics and needed parenteral nutrition. One case was managed conservatively, and the other required percutaneous nephrostomy. This particular case was a salvage procedure due to vesicoureteral obstruction after failed CSUR and the only patient in our series without a ureteral stent to protect the anastomosis. Similarly, Lashley et al. (19) reported a prolonged output from perianastomotic drains in 13% of patients from an average of 15 days (7–31 days). A shunt across the Y-junction, causing stasis, was described for extravesical bifid ureter (21). We observed no problems with yo-yo reflux in any of our patients. This is consistent with the studies referenced above, none of which reported any complication attributed to yo-yo reflux. Furthermore, Steyaert et al. (13), Storm et al., and Chandrasekharam et al. (22) did not observe perioperative or post-operative complications at 6, 8, or 19 months of mean follow-up.

The rate of post-operative complications was similar following both LUU and CSUR. In both of our groups, post-operative febrile UTIs occurred. However, these were only single events, and post-operative VCUG was therefore not performed. Owing to two complicated courses with anastomotic leaks and consecutive prolonged hospitalization (13 and 26 days), the mean hospitalization time after LUU was quite high (6.8 days), even higher than after CSUR (6.6 days). The effect of these outliers is eliminated when the median time of hospitalization is considered: this was shorter after LUU (5 days) than after CSUR (7 days). The mean hospital stay after LUU reported in the comparable literature is 3 days, only one prolonged hospitalization (7 days) due to pyelonephritis has been reported (11–14).

Operative time was longer in our first five cases using LUU but became similar to the operative time for CSUR thereafter. Our most recent 10 LUUs exhibit a clear learning curve, with a mean duration of 166 min including cystoscopy and repositioning of the patient from lithotomy to supine position. This operative time is similar to the 169 min for CSUR. González et al. (11) reported a mean operative time of 256 min, and Storm et al. (14) took 187 min mean, both including cystoscopy. The teams around Chandrasekharam et al. (22) and Steyaert et al. (13), who did not clearly include or exclude time for cystoscopy, both reported requiring 120 min per procedure in case series of eight and two patients, respectively. In summary, LUU offers the advantages of a minimally invasive procedure, resulting in smaller scars and shorter hospitalization times, without concerns about more complications or longer operation times than CSUR. Owing to the retrospective design of this study, certain points have to be commented. The goal of this study was to assess, whether in children with symptomatic duplex kidney anomalies, who would typically get scheduled for CSUR, a less invasive surgical approach could be a valid alternative. For this reason, we wanted to compare LUU to CSUR and not open ureteroureterostomy to CSUR.

For the same reason, we included patients with varying indications for surgery, even exceptional cases with excessive operative times such as a bilateral LUU (05:35 h) or a salvage procedure LUU with anastomosis to the contralateral ureter (08:15 h). Such a heterogeneous population with a different surgical risk profile is problematic in a retrospective study. Nevertheless, our study shows that LUU can be used as an alternative to CSUR irrespective of the indication for surgery.

Although LUU is not a novel method, we believe that the comparison with CSUR, the large case number with the extended follow-up, and the diversity of our population provides valuable information for pediatric urologists, especially considering the fact that there is little comparable literature.

## Conclusion

LUU is a safe and efficacious treatment option for children with duplex kidney anomalies and can be used as an alternative to CSUR.

## Data Availability Statement

The original contributions presented in the study are included in the article/supplementary material, further inquiries can be directed to the corresponding authors.

## Ethics Statement

The studies involving human participants were reviewed and approved by Ethics committee of the Canton Zurich (2019-00305). Written informed consent from the participants' legal guardian/next of kin was not required to participate in this study in accordance with the national legislation and the institutional requirements.

## Author Contributions

TG and LM contributed to structure, content, and writing of the manuscript. DW and RGn contributed to structure, content, and reviewed the manuscript. RGn evaluated the ultrasounds and reviewed the manuscript. All authors contributed to the article and approved the submitted version.

## Conflict of Interest

The authors declare that the research was conducted in the absence of any commercial or financial relationships that could be construed as a potential conflict of interest.

## Abbreviations

CSUR: common sheath ureteral reimplantation
LUU: laparoscopic ureteroureterostomy
UTI: urinary tract infections
VCUG: voiding cystourethrogram
VUR: vesicoureteral reflux.
